# *catena*-Poly[[[bis­(1,10-phenanthroline-κ^2^ *N*,*N*′)manganese(II)]-μ-9,10-dioxo­anthracene-1,5-disulfonato-κ^2^ *O* ^1^:*O* ^5^] tetra­hydrate]

**DOI:** 10.1107/S1600536809019503

**Published:** 2009-05-29

**Authors:** Jia Jia, Wen Feng, Hong-Kun Zhao, En-Cui Yang

**Affiliations:** a College of Chemistry and Life Science, Tianjin Key Laboratory of Structure and Performance of Functional Molecule, Tianjin Normal University, Tianjin 300387, People’s Republic of China

## Abstract

The title complex, {[Mn(C_14_H_6_O_8_S_2_)(C_12_H_8_N_2_)_2_]·4H_2_O}_*n*_, exhibits a chain-like polymeric structure with 9,10-dioxo­anthracene-1,5-disulfonate anions bridging Mn^II^ atoms in a bis-monodentate mode. The unique Mn^II^ atom is located on a crystallographic centre of inversion. Four N atoms from two chelating 1,10-phenanthroline ligands and two sulfonate O atoms from two symmetry-related 9,10-dioxoanthracene-1,5-disulfonate anions give rise to a slightly distorted octa­hedral coordination environment around the Mn^II^ centre. The centroid of the central ring of the anthraquinone ligand represents another crystallographic centre of inversion. In the crystal structure, inter­ligand π–π stacking [centroid-to-centroid distances 3.532 (1) and 3.497 (3) Å] and inter­molecular O—H⋯O hydrogen-bonding inter­actions assemble the chains into a three-dimensional supra­molecular network.

## Related literature

For applications of organosulfonate-based metal complexes, see: Côté & Shimizu (2003 ▶); Cai (2004 ▶). For synthetic procedure, see: Cui *et al.* (2007 ▶); Dai *et al.* (2006 ▶); Zhao *et al.* (2007 ▶). For related structures, see: Cai *et al.* (2001 ▶); Du *et al.* (2006 ▶); Gándara *et al.* (2006 ▶); Wu *et al.* (2007 ▶).
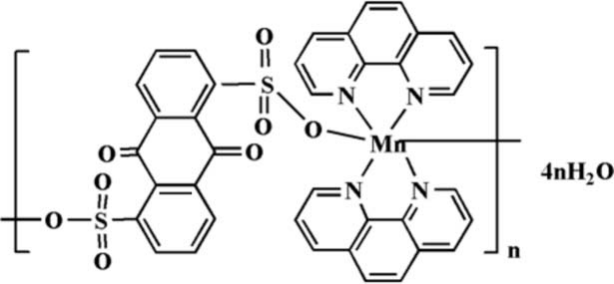


## Experimental

### 

#### Crystal data

[Mn(C_14_H_6_O_8_S_2_)(C_12_H_8_N_2_)_2_]·4H_2_O
*M* *_r_* = 853.74
Triclinic, 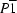

*a* = 8.8882 (9) Å
*b* = 9.578 (1) Å
*c* = 11.016 (1) Å
α = 105.962 (1)°
β = 103.050 (1)°
γ = 93.120 (1)°
*V* = 871.5 (2) Å^3^
*Z* = 1
Mo *K*α radiation
μ = 0.58 mm^−1^
*T* = 294 K
0.32 × 0.28 × 0.26 mm

#### Data collection

Bruker APEXII CCD area-detector diffractometer
Absorption correction: multi-scan (*SADABS*; Sheldrick, 1996 ▶) *T*_min_ = 0.838, *T*_max_ = 0.865
4767 measured reflections
3042 independent reflections
2751 reflections with *I* > 2σ(*I*)
*R*_int_ = 0.011

#### Refinement

*R*[*F* ^2^ > 2σ(*F* ^2^)] = 0.030
*wR*(*F* ^2^) = 0.086
*S* = 1.05
3042 reflections
259 parameters
H-atom parameters constrained
Δρ_max_ = 0.36 e Å^−3^
Δρ_min_ = −0.32 e Å^−3^

### 

Data collection: *APEX2* (Bruker, 2003 ▶); cell refinement: *SAINT* (Bruker, 2001 ▶); data reduction: *SAINT*; program(s) used to solve structure: *SHELXS97* (Sheldrick, 2008 ▶); program(s) used to refine structure: *SHELXL97* (Sheldrick, 2008 ▶); molecular graphics: *SHELXTL* (Sheldrick, 2008 ▶) and *DIAMOND* (Brandenburg & Berndt, 1999 ▶); software used to prepare material for publication: *SHELXTL*.

## Supplementary Material

Crystal structure: contains datablocks I, global. DOI: 10.1107/S1600536809019503/im2118sup1.cif

Structure factors: contains datablocks I. DOI: 10.1107/S1600536809019503/im2118Isup2.hkl

Additional supplementary materials:  crystallographic information; 3D view; checkCIF report

## Figures and Tables

**Table 1:** Hydrogen-bond geometry (Å, °)

*D*—H⋯*A*	*D*—H	H⋯*A*	*D*⋯*A*	*D*—H⋯*A*
O5*W*—H5*A*⋯O2^i^	0.85	2.03	2.826 (2)	156
O5*W*—H5*B*⋯O2^ii^	0.85	2.10	2.948 (2)	172
O6*W*—H6*A*⋯O5*W*^iii^	0.85	2.13	2.868 (3)	145
O6*W*—H6*B*⋯O3^iv^	0.85	2.12	2.922 (3)	157

Symmetry codes: (i) 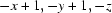; (ii) 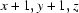; (iii) 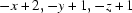; (iv) 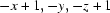.

**Fig. 1.**
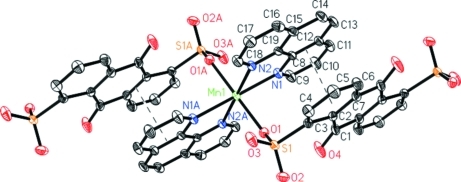
The local coordination environment of MnII in I) drawn with 30% probability displacement ellipsoids. H atoms were omitted for clarity. The short dashed lines indicate interligand π-π stacking interactions [Symmetry code: (A) 1 - x, 1 - y, 1 - z.]

**Fig. 2.**
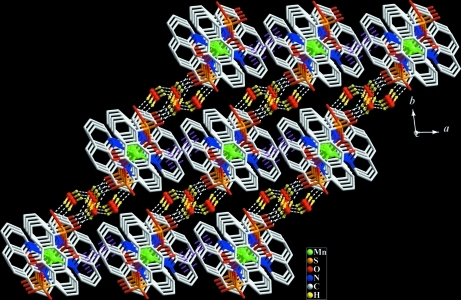
The three-dimensional supramolecular network of (I) produced by hydrogen-bonding and π–π stacking interactions.

## supplementary crystallographic information

### Comment

Recently, organosulfonate-based metal complexes have drawn intense interest due to their adjustable coordination ability and interesting applications as functional materials [Cai, 2004; Côté & Shimizu, 2003; Zhao *et al.*, 2007]. By introducing popular nitrogen-containing functional organic molecules as coligands, a series of sulfonate-based complexes have successfully been synthesized, which exhibit diverse structures ranging from discrete zero-dimensional (0D) to infinite high-dimensional structures [Cai *et al.*, 2001; Gándara *et al.*, 2006; Du *et al.*, 2006]. As part of our continuous investigation on the coordination chemistry of mixed-ligand systems [Dai *et al.*, 2006; Cui *et al.*, 2007; Wu *et al.*, 2007], we herein report the crystal structure of a Mn^II^ complex with 1,10-phenanthroline and 9,10-dioxoanthracene-1,5-disulfonate ligands (I).

The local coordination environment of Mn^II^ atom in I is shown in Fig. 1. The unique Mn^II^ atom is situated on a crystallodraphic centre of inversion and is six-coordinated by four N atoms from two chelating 1,10-phenanthroline ligands and two sulfonate O atoms from two independent 9,10-dioxoanthracene-1,5-disulfonate anions, exhibiting a slightly distorted octahedral coordination mode. The centrosymmetric 9,10-dioxoanthracene-1,5-disulfonate anion adopts a bis-monodentate mode, linking the adjacent Mn^II^ atoms into a one-dimensional infinite chain along the *c*-axis (Fig. 2). Two interligand π–π stacking interactions between the intrachain 1,10-phenanthroline and anthraquinone ring as well as between the two interchain 1,10-phenanthroline rings were observed (Fig. 1 and 2), which stabilize the one-dimensional chain and further extend the chains into a two-dimensional plane. The centroid–centroid distance and the dihedral angle between the 1,10-phenanthroline and anthraquinone ring measures to 3.532 (1) Å and 2.704 (4)°. In contrast, the π-stacking parameters between the interchain 1,10-phenanthroline rings are 3.497 (3) Å and 0.0°, respectively.

Additionally, the adjacent two-dimensional planes are extended into a three-dimensional supramolecular network by fourfold O—H···O hydrogen-bonding interactions between the sulfonate O atoms and the lattice water molecules (Table 1 and Fig. 2).

### Experimental

A mixture of disodium 9,10-dioxoanthracene-1,5-disulfonate (164.8 mg, 0.4 mmol), Mn(OAc)_2_.4H_2_O (98.0 mg, 0.4 mmol), 1,10-phenanthroline (79.3 mg, 0.4 mmol), and H_2_O (20 ml) was sealed in a 23 ml teflon lined stainless steel vessel. The vessel was heated to 413 K for 2 d under autogenous pressure and then cooled to room temperature at a rate of 2.4 K/h. Yellow block-shaped crystals suitable for X-ray analysis were obtained in a 41% yield. Analysis calculated for C_19_H_15_Mn_0.50_N_2_O_6_S: C 53.46, H 3.54, N 6.56%; found: C 53.56, H 3.50, N 6.70%.

### Refinement

H atoms were located from difference Fourier maps, but were subsequently placed in calculated positions and treated as riding, with C—H = 0.93 Å and O—H = 0.85 Å. All H atoms were allocated displacement parameters related to those of their parent atoms [*U*_iso_(H) = 1.2Ueq(C,O)].

### Crystal data

**Table:**

[Mn(C_14_H_6_O_8_S_2_)(C_12_H_8_N_2_)_2_]·4H_2_O	*Z* = 1
*M**_r_* = 853.74	*F*(000) = 439
Triclinic, *P*1	*D*_x_ = 1.627 Mg m^−^^3^
Hall symbol: -P 1	Mo *K*α radiation, λ = 0.71073 Å
*a* = 8.8882 (9) Å	Cell parameters from 3691 reflections
*b* = 9.578 (1) Å	θ = 2.2–27.9°
*c* = 11.016 (1) Å	µ = 0.57 mm^−^^1^
α = 105.962 (1)°	*T* = 294 K
β = 103.050 (1)°	Block, yellow
γ = 93.120 (1)°	0.32 × 0.28 × 0.26 mm
*V* = 871.5 (2) Å^3^	

### Data collection

**Table:**

Bruker APEXII CCD area-detector diffractometer	3042 independent reflections
Radiation source: fine-focus sealed tube	2751 reflections with *I* > 2σ(*I*)
graphite	*R*_int_ = 0.011
φ and ω scans	θ_max_ = 25.0°, θ_min_ = 2.0°
Absorption correction: multi-scan (*SADABS*; Sheldrick, 1996)	*h* = −9→10
*T*_min_ = 0.838, *T*_max_ = 0.865	*k* = −11→8
4767 measured reflections	*l* = −13→13

### Refinement

**Table:**

Refinement on *F*^2^	Primary atom site location: structure-invariant direct methods
Least-squares matrix: full	Secondary atom site location: difference Fourier map
*R*[*F*^2^ > 2σ(*F*^2^)] = 0.030	Hydrogen site location: inferred from neighbouring sites
*wR*(*F*^2^) = 0.086	H-atom parameters constrained
*S* = 1.05	*w* = 1/[σ^2^(*F*_o_^2^) + (0.0505*P*)^2^ + 0.3551*P*] where *P* = (*F*_o_^2^ + 2*F*_c_^2^)/3
3042 reflections	(Δ/σ)_max_ < 0.001
259 parameters	Δρ_max_ = 0.36 e Å^−^^3^
0 restraints	Δρ_min_ = −0.32 e Å^−^^3^

### Special details

**Table:**

Geometry. All e.s.d.'s (except the e.s.d. in the dihedral angle between two l.s. planes) are estimated using the full covariance matrix. The cell e.s.d.'s are taken into account individually in the estimation of e.s.d.'s in distances, angles and torsion angles; correlations between e.s.d.'s in cell parameters are only used when they are defined by crystal symmetry. An approximate (isotropic) treatment of cell e.s.d.'s is used for estimating e.s.d.'s involving l.s. planes.
Refinement. Refinement of *F*^2^ against ALL reflections. The weighted *R*-factor *wR* and goodness of fit *S* are based on *F*^2^, conventional *R*-factors *R* are based on *F*, with *F* set to zero for negative *F*^2^. The threshold expression of *F*^2^ > σ(*F*^2^) is used only for calculating *R*-factors(gt) *etc*. and is not relevant to the choice of reflections for refinement. *R*-factors based on *F*^2^ are statistically about twice as large as those based on *F*, and *R*- factors based on ALL data will be even larger.

### Fractional atomic coordinates and isotropic or equivalent isotropic displacement parameters (Å^2^)

**Table:**

	*x*	*y*	*z*	*U*_iso_*/*U*_eq_	
Mn1	0.5000	0.5000	0.5000	0.02553 (13)	
S1	0.34382 (5)	0.24029 (5)	0.21379 (4)	0.03110 (14)	
O1	0.34939 (15)	0.38120 (14)	0.30894 (12)	0.0344 (3)	
O2	0.19137 (17)	0.18841 (18)	0.12816 (15)	0.0506 (4)	
O3	0.40672 (19)	0.13426 (17)	0.27693 (17)	0.0532 (4)	
O4	0.2494 (2)	0.4691 (2)	0.0881 (2)	0.0710 (6)	
N1	0.59998 (18)	0.65125 (17)	0.40181 (15)	0.0323 (3)	
N2	0.71074 (17)	0.40323 (16)	0.44201 (15)	0.0294 (3)	
C1	0.3690 (2)	0.4857 (2)	0.05604 (19)	0.0388 (5)	
C2	0.4839 (2)	0.3789 (2)	0.05903 (17)	0.0307 (4)	
C3	0.4760 (2)	0.2649 (2)	0.11671 (17)	0.0306 (4)	
C4	0.5807 (2)	0.1633 (2)	0.1047 (2)	0.0389 (5)	
H4	0.5756	0.0884	0.1429	0.047*	
C5	0.6932 (3)	0.1712 (2)	0.0368 (2)	0.0448 (5)	
H5	0.7597	0.0995	0.0264	0.054*	
C6	0.7061 (2)	0.2844 (2)	−0.0148 (2)	0.0425 (5)	
H6	0.7838	0.2916	−0.0576	0.051*	
C7	0.6032 (2)	0.3891 (2)	−0.00353 (18)	0.0338 (4)	
C8	0.7230 (2)	0.60542 (19)	0.35365 (17)	0.0284 (4)	
C9	0.5491 (3)	0.7727 (2)	0.3818 (2)	0.0454 (5)	
H9	0.4652	0.8050	0.4142	0.055*	
C10	0.6133 (3)	0.8547 (2)	0.3155 (2)	0.0486 (5)	
H10	0.5727	0.9391	0.3041	0.058*	
C11	0.7358 (3)	0.8100 (2)	0.2677 (2)	0.0448 (5)	
H11	0.7806	0.8639	0.2235	0.054*	
C12	0.7950 (2)	0.6821 (2)	0.28506 (19)	0.0351 (4)	
C13	0.9234 (2)	0.6277 (2)	0.2369 (2)	0.0436 (5)	
H13	0.9702	0.6779	0.1911	0.052*	
C14	0.9780 (2)	0.5056 (2)	0.2565 (2)	0.0435 (5)	
H14	1.0616	0.4724	0.2239	0.052*	
C15	0.9090 (2)	0.4258 (2)	0.32685 (19)	0.0345 (4)	
C16	0.9640 (2)	0.2989 (2)	0.3505 (2)	0.0419 (5)	
H16	1.0480	0.2634	0.3200	0.050*	
C17	0.8938 (2)	0.2278 (2)	0.4183 (2)	0.0431 (5)	
H17	0.9290	0.1436	0.4348	0.052*	
C18	0.7684 (2)	0.2839 (2)	0.4623 (2)	0.0383 (5)	
H18	0.7217	0.2349	0.5090	0.046*	
C19	0.7815 (2)	0.47471 (19)	0.37489 (17)	0.0281 (4)	
O5W	0.9995 (2)	0.9273 (2)	0.12804 (18)	0.0708 (5)	
H5A	0.9520	0.8694	0.0539	0.106*	
H5B	1.0469	1.0073	0.1289	0.106*	
O6W	0.7867 (3)	0.0448 (3)	0.6265 (2)	0.0998 (8)	
H6A	0.8658	0.0844	0.6892	0.150*	
H6B	0.7305	−0.0255	0.6340	0.150*	

### Atomic displacement parameters (Å^2^)

**Table:**

	*U*^11^	*U*^22^	*U*^33^	*U*^12^	*U*^13^	*U*^23^
Mn1	0.0264 (2)	0.0268 (2)	0.0277 (2)	0.00381 (15)	0.01252 (15)	0.01015 (15)
S1	0.0332 (3)	0.0287 (2)	0.0334 (3)	−0.00064 (18)	0.0137 (2)	0.00874 (19)
O1	0.0354 (7)	0.0363 (7)	0.0301 (7)	0.0033 (5)	0.0105 (6)	0.0061 (5)
O2	0.0369 (8)	0.0589 (10)	0.0456 (9)	−0.0125 (7)	0.0110 (7)	0.0018 (7)
O3	0.0627 (10)	0.0451 (9)	0.0749 (11)	0.0172 (7)	0.0383 (9)	0.0358 (8)
O4	0.0518 (10)	0.1099 (15)	0.1036 (15)	0.0435 (10)	0.0535 (10)	0.0824 (13)
N1	0.0336 (8)	0.0312 (8)	0.0380 (9)	0.0052 (6)	0.0160 (7)	0.0138 (7)
N2	0.0286 (8)	0.0310 (8)	0.0330 (8)	0.0038 (6)	0.0120 (6)	0.0128 (6)
C1	0.0334 (10)	0.0589 (13)	0.0369 (11)	0.0142 (9)	0.0180 (8)	0.0259 (10)
C2	0.0282 (9)	0.0396 (10)	0.0251 (9)	0.0035 (8)	0.0082 (7)	0.0095 (8)
C3	0.0305 (9)	0.0329 (9)	0.0269 (9)	0.0003 (7)	0.0098 (7)	0.0046 (7)
C4	0.0430 (11)	0.0329 (10)	0.0435 (11)	0.0057 (8)	0.0177 (9)	0.0100 (9)
C5	0.0461 (12)	0.0413 (11)	0.0546 (13)	0.0159 (9)	0.0245 (10)	0.0148 (10)
C6	0.0395 (11)	0.0510 (12)	0.0467 (12)	0.0136 (9)	0.0250 (10)	0.0171 (10)
C7	0.0304 (9)	0.0446 (11)	0.0308 (10)	0.0083 (8)	0.0123 (8)	0.0137 (8)
C8	0.0267 (9)	0.0315 (9)	0.0269 (9)	−0.0024 (7)	0.0092 (7)	0.0074 (7)
C9	0.0479 (12)	0.0411 (11)	0.0634 (14)	0.0145 (9)	0.0302 (11)	0.0265 (10)
C10	0.0555 (14)	0.0404 (11)	0.0653 (15)	0.0115 (10)	0.0261 (12)	0.0304 (11)
C11	0.0519 (13)	0.0416 (11)	0.0509 (12)	−0.0002 (10)	0.0215 (10)	0.0240 (10)
C12	0.0357 (10)	0.0366 (10)	0.0346 (10)	−0.0039 (8)	0.0121 (8)	0.0120 (8)
C13	0.0407 (11)	0.0512 (12)	0.0470 (12)	−0.0032 (9)	0.0247 (10)	0.0180 (10)
C14	0.0362 (11)	0.0507 (12)	0.0494 (12)	0.0034 (9)	0.0246 (10)	0.0132 (10)
C15	0.0284 (9)	0.0385 (10)	0.0359 (10)	0.0016 (8)	0.0132 (8)	0.0062 (8)
C16	0.0315 (10)	0.0453 (12)	0.0501 (12)	0.0103 (9)	0.0180 (9)	0.0089 (10)
C17	0.0405 (11)	0.0395 (11)	0.0550 (13)	0.0138 (9)	0.0162 (10)	0.0182 (10)
C18	0.0366 (10)	0.0396 (11)	0.0482 (12)	0.0095 (8)	0.0182 (9)	0.0211 (9)
C19	0.0248 (9)	0.0314 (9)	0.0261 (9)	−0.0012 (7)	0.0075 (7)	0.0055 (7)
O5W	0.0725 (12)	0.0829 (13)	0.0555 (10)	−0.0218 (10)	0.0079 (9)	0.0305 (10)
O6W	0.1002 (17)	0.1057 (18)	0.0955 (16)	−0.0265 (14)	−0.0050 (13)	0.0642 (14)

### Geometric parameters (Å, °)

**Table:**

Mn1—O1^i^	2.1820 (12)	C7—C1^ii^	1.499 (3)
Mn1—O1	2.1820 (12)	C8—C12	1.412 (3)
Mn1—N2	2.2758 (15)	C8—C19	1.438 (3)
Mn1—N2^i^	2.2758 (15)	C9—C10	1.390 (3)
Mn1—N1^i^	2.2834 (15)	C9—H9	0.9300
Mn1—N1	2.2834 (15)	C10—C11	1.353 (3)
S1—O2	1.4368 (15)	C10—H10	0.9300
S1—O3	1.4499 (16)	C11—C12	1.403 (3)
S1—O1	1.4539 (13)	C11—H11	0.9300
S1—C3	1.8042 (18)	C12—C13	1.429 (3)
O4—C1	1.210 (2)	C13—C14	1.343 (3)
N1—C9	1.326 (3)	C13—H13	0.9300
N1—C8	1.362 (2)	C14—C15	1.432 (3)
N2—C18	1.331 (2)	C14—H14	0.9300
N2—C19	1.361 (2)	C15—C16	1.402 (3)
C1—C2	1.485 (3)	C15—C19	1.406 (3)
C1—C7^ii^	1.499 (3)	C16—C17	1.362 (3)
C2—C7	1.401 (2)	C16—H16	0.9300
C2—C3	1.412 (3)	C17—C18	1.390 (3)
C3—C4	1.383 (3)	C17—H17	0.9300
C4—C5	1.388 (3)	C18—H18	0.9300
C4—H4	0.9300	O5W—H5A	0.8502
C5—C6	1.366 (3)	O5W—H5B	0.8502
C5—H5	0.9300	O6W—H6A	0.8503
C6—C7	1.393 (3)	O6W—H6B	0.8500
C6—H6	0.9300		
			
O1^i^—Mn1—O1	180.0	C5—C6—H6	119.9
O1^i^—Mn1—N2	88.86 (5)	C7—C6—H6	119.9
O1—Mn1—N2	91.14 (5)	C6—C7—C2	120.69 (18)
O1^i^—Mn1—N2^i^	91.14 (5)	C6—C7—C1^ii^	116.82 (17)
O1—Mn1—N2^i^	88.86 (5)	C2—C7—C1^ii^	122.43 (17)
N2—Mn1—N2^i^	180.00 (7)	N1—C8—C12	122.37 (17)
O1^i^—Mn1—N1^i^	87.76 (5)	N1—C8—C19	118.36 (15)
O1—Mn1—N1^i^	92.24 (5)	C12—C8—C19	119.27 (16)
N2—Mn1—N1^i^	106.48 (5)	N1—C9—C10	124.2 (2)
N2^i^—Mn1—N1^i^	73.52 (5)	N1—C9—H9	117.9
O1^i^—Mn1—N1	92.24 (5)	C10—C9—H9	117.9
O1—Mn1—N1	87.76 (5)	C11—C10—C9	119.0 (2)
N2—Mn1—N1	73.52 (5)	C11—C10—H10	120.5
N2^i^—Mn1—N1	106.48 (5)	C9—C10—H10	120.5
N1^i^—Mn1—N1	179.999 (2)	C10—C11—C12	119.77 (19)
O2—S1—O3	112.74 (10)	C10—C11—H11	120.1
O2—S1—O1	112.54 (9)	C12—C11—H11	120.1
O3—S1—O1	111.06 (9)	C11—C12—C8	117.56 (18)
O2—S1—C3	108.28 (9)	C11—C12—C13	122.97 (18)
O3—S1—C3	104.66 (9)	C8—C12—C13	119.46 (19)
O1—S1—C3	107.04 (8)	C14—C13—C12	121.33 (19)
S1—O1—Mn1	135.36 (8)	C14—C13—H13	119.3
C9—N1—C8	117.14 (16)	C12—C13—H13	119.3
C9—N1—Mn1	128.11 (13)	C13—C14—C15	120.80 (19)
C8—N1—Mn1	114.68 (12)	C13—C14—H14	119.6
C18—N2—C19	117.16 (16)	C15—C14—H14	119.6
C18—N2—Mn1	127.72 (12)	C16—C15—C19	117.87 (18)
C19—N2—Mn1	115.07 (12)	C16—C15—C14	122.40 (18)
O4—C1—C2	121.34 (19)	C19—C15—C14	119.73 (19)
O4—C1—C7^ii^	119.27 (19)	C17—C16—C15	119.72 (18)
C2—C1—C7^ii^	119.26 (16)	C17—C16—H16	120.1
C7—C2—C3	118.43 (17)	C15—C16—H16	120.1
C7—C2—C1	117.76 (17)	C16—C17—C18	118.60 (19)
C3—C2—C1	123.77 (16)	C16—C17—H17	120.7
C4—C3—C2	119.42 (17)	C18—C17—H17	120.7
C4—C3—S1	114.57 (15)	N2—C18—C17	124.23 (18)
C2—C3—S1	125.96 (14)	N2—C18—H18	117.9
C3—C4—C5	121.22 (19)	C17—C18—H18	117.9
C3—C4—H4	119.4	N2—C19—C15	122.41 (17)
C5—C4—H4	119.4	N2—C19—C8	118.19 (15)
C6—C5—C4	119.86 (19)	C15—C19—C8	119.40 (16)
C6—C5—H5	120.1	H5A—O5W—H5B	117.0
C4—C5—H5	120.1	H6A—O6W—H6B	117.0
C5—C6—C7	120.25 (18)		
			
O2—S1—O1—Mn1	157.33 (11)	C4—C5—C6—C7	−2.3 (3)
O3—S1—O1—Mn1	29.85 (14)	C5—C6—C7—C2	−0.9 (3)
C3—S1—O1—Mn1	−83.83 (12)	C5—C6—C7—C1^ii^	176.6 (2)
O1^i^—Mn1—O1—S1	163 (13)	C3—C2—C7—C6	3.5 (3)
N2—Mn1—O1—S1	33.35 (11)	C1—C2—C7—C6	−174.07 (19)
N2^i^—Mn1—O1—S1	−146.65 (11)	C3—C2—C7—C1^ii^	−173.82 (17)
N1^i^—Mn1—O1—S1	−73.20 (11)	C1—C2—C7—C1^ii^	8.6 (3)
N1—Mn1—O1—S1	106.81 (11)	C9—N1—C8—C12	0.0 (3)
O1^i^—Mn1—N1—C9	−91.40 (18)	Mn1—N1—C8—C12	177.27 (14)
O1—Mn1—N1—C9	88.60 (18)	C9—N1—C8—C19	179.47 (18)
N2—Mn1—N1—C9	−179.54 (19)	Mn1—N1—C8—C19	−3.2 (2)
N2^i^—Mn1—N1—C9	0.45 (19)	C8—N1—C9—C10	0.0 (3)
N1^i^—Mn1—N1—C9	−37 (8)	Mn1—N1—C9—C10	−176.90 (18)
O1^i^—Mn1—N1—C8	91.68 (13)	N1—C9—C10—C11	−0.1 (4)
O1—Mn1—N1—C8	−88.32 (13)	C9—C10—C11—C12	0.4 (4)
N2—Mn1—N1—C8	3.53 (12)	C10—C11—C12—C8	−0.4 (3)
N2^i^—Mn1—N1—C8	−176.47 (12)	C10—C11—C12—C13	179.7 (2)
N1^i^—Mn1—N1—C8	146 (8)	N1—C8—C12—C11	0.2 (3)
O1^i^—Mn1—N2—C18	86.18 (16)	C19—C8—C12—C11	−179.24 (17)
O1—Mn1—N2—C18	−93.82 (16)	N1—C8—C12—C13	−179.86 (18)
N2^i^—Mn1—N2—C18	36 (17)	C19—C8—C12—C13	0.7 (3)
N1^i^—Mn1—N2—C18	−1.15 (17)	C11—C12—C13—C14	179.3 (2)
N1—Mn1—N2—C18	178.85 (17)	C8—C12—C13—C14	−0.6 (3)
O1^i^—Mn1—N2—C19	−96.18 (12)	C12—C13—C14—C15	−0.2 (3)
O1—Mn1—N2—C19	83.82 (12)	C13—C14—C15—C16	−179.2 (2)
N2^i^—Mn1—N2—C19	−146 (17)	C13—C14—C15—C19	0.8 (3)
N1^i^—Mn1—N2—C19	176.49 (12)	C19—C15—C16—C17	−0.1 (3)
N1—Mn1—N2—C19	−3.51 (12)	C14—C15—C16—C17	179.9 (2)
O4—C1—C2—C7	167.4 (2)	C15—C16—C17—C18	0.1 (3)
C7^ii^—C1—C2—C7	−8.3 (3)	C19—N2—C18—C17	−0.8 (3)
O4—C1—C2—C3	−10.0 (3)	Mn1—N2—C18—C17	176.82 (16)
C7^ii^—C1—C2—C3	174.24 (17)	C16—C17—C18—N2	0.4 (3)
C7—C2—C3—C4	−3.0 (3)	C18—N2—C19—C15	0.7 (3)
C1—C2—C3—C4	174.46 (18)	Mn1—N2—C19—C15	−177.20 (13)
C7—C2—C3—S1	174.29 (14)	C18—N2—C19—C8	−178.94 (17)
C1—C2—C3—S1	−8.3 (3)	Mn1—N2—C19—C8	3.2 (2)
O2—S1—C3—C4	−108.81 (16)	C16—C15—C19—N2	−0.3 (3)
O3—S1—C3—C4	11.67 (17)	C14—C15—C19—N2	179.75 (18)
O1—S1—C3—C4	129.62 (14)	C16—C15—C19—C8	179.36 (17)
O2—S1—C3—C2	73.81 (18)	C14—C15—C19—C8	−0.6 (3)
O3—S1—C3—C2	−165.72 (16)	N1—C8—C19—N2	0.1 (2)
O1—S1—C3—C2	−47.77 (18)	C12—C8—C19—N2	179.57 (16)
C2—C3—C4—C5	−0.2 (3)	N1—C8—C19—C15	−179.58 (16)
S1—C3—C4—C5	−177.72 (16)	C12—C8—C19—C15	−0.1 (3)
C3—C4—C5—C6	2.8 (3)		

Symmetry codes: (i) −*x*+1, −*y*+1, −*z*+1; (ii) −*x*+1, −*y*+1, −*z*.

### Hydrogen-bond geometry (Å, °)

**Table:**

*D*—H···*A*	*D*—H	H···*A*	*D*···*A*	*D*—H···*A*
O5W—H5A···O2^ii^	0.85	2.03	2.826 (2)	156
O5W—H5B···O2^iii^	0.85	2.10	2.948 (2)	172
O6W—H6A···O5W^iv^	0.85	2.13	2.868 (3)	145
O6W—H6B···O3^v^	0.85	2.12	2.922 (3)	157

Symmetry codes: (ii) −*x*+1, −*y*+1, −*z*; (iii) *x*+1, *y*+1, *z*; (iv) −*x*+2, −*y*+1, −*z*+1; (v) −*x*+1, −*y*, −*z*+1.
